# The Effectiveness of Traditional Chinese Yijinjing Qigong Exercise for the Patients With Knee Osteoarthritis on the Pain, Dysfunction, and Mood Disorder: A Pilot Randomized Controlled Trial

**DOI:** 10.3389/fmed.2021.792436

**Published:** 2022-01-11

**Authors:** Shuaipan Zhang, Guangxin Guo, Xing Li, Fei Yao, Zhiwei Wu, Qingguang Zhu, Min Fang

**Affiliations:** ^1^School of Acupuncture-Moxibustion and Tuina, Shanghai University of Traditional Chinese Medicine, Shanghai, China; ^2^Tuina Department, Yue Yang Hospital of Integrated Traditional Chinese and Western Medicine, Shanghai University of Traditional Chinese Medicine, Shanghai, China; ^3^Department of Integrated Traditional Chinese and Western Medicine, Institute of Integrated Traditional Chinese and Western Medicine, Shanghai University of Traditional Chinese Medicine, Shanghai, China; ^4^Tuina Department, Institute of Tuina, Shanghai University of Traditional Chinese Medicine, Shanghai, China

**Keywords:** knee osteoarthritis, Yijinjing Qigong Exercise, stretching training exercises, efficacy, randomized controlled trial

## Abstract

**Background:** Although traditional Chinese Yijinjing Qigong Exercise (YJJQE) is popularly used in China, to alleviate symptoms of people with knee osteoarthritis (KOA), no randomized controlled trials (RCTs) are available to evaluate the effects of YJJQE in patients with KOA. The purpose of this trial is to assess the clinical efficacy of YJJQE for patients with KOA.

**Methods:** A total of 50 participants clinically diagnosed with KOA are randomly (1:1) assigned to the YJJQE group (*n* = 25) and to the stretching training exercise (STE) group (*n* = 25), for a 40-min exercise session twice a week for 12 weeks. All outcome measures are collected at baseline and at 12-week ending intervention, which includes the primary outcomes of Western Ontario and McMaster Universities Osteoarthritis Index Scale (WOMAC), the secondary outcomes of visual analog scale (VAS), mental component summary (MCS), physical component summary (PCS), Beck depression inventory (BDI), perceived stress scale (PSS), Berg balance scale (BBS), and the Gait functional mobility data.

**Results:** The YJJQE group did not have any significant changes compared to the control group on the WOMAC score after the 12-week intervention (*P* > 0.05), though the YJJQE group demonstrated better performance in MCS, BDI, and PSS (*P* = 0.002, *P* = 0.001, and *P* = 0.026, respectively) than the control group. No serious adverse events occurred in either group, and only mild muscle soreness was reported during both exercise treatments.

**Conclusion:** Because no difference between both groups was shown, with regards to the primary outcome measurement (WOMAC), it can hardly explain that the YJJQE had an advantageous effect on patients experiencing the pain and dysfunction of knee osteoarthritis. However, compared to the control group, YJJQE appeared to be associated with improvements in psychological well-being including reduced stress, anxiety, depression, and mood disturbance to manage KOA. Further trials with larger sample sizes and follow-up studies will be required.

**Clinical Trial Registration:** https://www.chictr.org.cn/edit.aspx?pid=60357&htm=4, ChiCTR2000037256.

## Introduction

Knee osteoarthritis (KOA) accounts for ~85% of the worldwide burden of osteoarthritis (1), a major age-related and public health problem that can cause pain, functional limitations, disability, and reduced quality of life. Approximately 27 million people in the United States suffer from osteoarthritis (2). With the increase in population, aging, and obesity, the prevalence of KOA also increases, as well as the burden of disease in healthcare services worldwide (3, 4). Currently, no effective approach is available to prevent the progression of the two major clinical problems, namely, disease pain and dysfunction. Oral antipyretic, analgesic, and non-steroidal anti-inflammatory drugs, as well as opioids, were associated with evident side effects, such as clinical gastrointestinal reactions, drug resistance, and addiction (5, 6). Joint cavity injection therapy is widely used, but the clinical efficacy is still controversial; excessive use of hormones may also lead to the risk of steroid arthropathy (7). Improving symptoms with prevention programmers is key to minimizing the social burden of KOA (8, 9). Exercise therapy, as an active treatment recommended by the American Rheumatology Guidelines with strong evidence (10), is increasingly used by clinical staff for the prevention and treatment of KOA. Recent trials have demonstrated that patients with KOA have experienced reduced pain and improved function after stretching training exercises (STE) (11). Yijinjing Qigong Exercise (YJJQE) is a multicomponent and traditional Chinese psychosomatic therapy that combines meditation with slow, gentle, stretching muscle movements, deep diaphragmatic breathing, and relaxation; it has been preliminarily proven to be beneficial for patients with KOA in some clinical research (12–14). However, no RCTs have been specifically conducted, using YJJQE, to evaluate its therapeutic benefits for those with KOA. To fill these gaps, we designed an RCT to determine the efficacy of YJJQE intervention to pain, functional mobility, and psychological well-being outcomes with KOA. Based on prior research, we hypothesized that YJJQE intervention has a better efficacy to improve functional mobility and to reduce pain symptomatology in patients with KOA.

## Materials and Methods

### Trial Design

The study used a parallel-group RCT design to compare an YJJQE intervention with an STE control across a 12-week period, which was conducted in Yue Yang Hospital of Integrated Traditional Chinese and Western Medicine affiliated with Shanghai University of Traditional Chinese Medicine. The study results and procedures were reported according to the CONSORT checklist. A total of 50 patients, who qualified to the inclusion criteria, had been recruited and randomly assigned to YJJQE group and STE group with a 1:1 ratio. Both exercise interventions are performed twice a week for 12 consecutive weeks, and all outcome measurements are performed at baseline and within the timeline. This trial protocol has been approved by the Ethics Committee of Yue Yang Hospital of Integrated Traditional Chinese and Western Medicine, which is affiliated with Shanghai University of Traditional Chinese Medicine (project number: KYSKSB-2020-138) and is registered in the Chinese Clinical Trial Registry (ChiCTR2000037256).

### Participants

Subjects were recruited mainly through online social platform advertising and hospital out-patient service from September 2020 to April 2021. We obtained an informed consent prior to baseline assessments for eligibility. Participants were required to satisfy the following criteria: (1) persons aged between 45 and 65, regardless of gender; (2) diagnostic criteria were in accordance with “Guidelines for the Diagnosis and Treatment of Osteoarthritis 2018 Edition.” Subjects were required to have the following pain symptoms, such as flat-surfaced walking, walking up and down stairs, standing up, and lying down, in the past month; (3) Kellgren/Lawrence was graded 1–2; (4) Body mass index ≤36 kg/m^2^; (5) all participants were examined based on pain and dysfunction by an orthopedic surgeon for inclusion; lastly (6) Visual analog scale (VAS) ≥2 points (0–10 points). The exclusion criteria were as follows: (1) YJJQE and stretching training experience in the last 3 months; (2) having the following serious diseases: severe coronary heart disease, heart failure, hypertension, cerebral infarction, cerebral hemorrhage, epilepsy, liver or kidney dysfunction, active bleeding, and cancer; (3) Recent one month history of segmental injection and surgery; (4) unable to independently complete the gait analysis test; (5) cognitive status evaluation score is below 24; lastly (6) unable to commit to exercise intervention and indicator collection as required by the protocol.

### Randomization, Allocation Concealment, and Blinding

Statistical software was used to generate a list of random numbers, and the third-party personnel has placed the digital numbers in opaque envelopes, marked it with time and signature, then unified it to the research coordinator. The research coordinator was responsible for directly recruiting potential subjects and randomly grouping them. Physical function assessors and data managers were unaware of the treatment assignments during enrollment and follow-up observations. All statistical analyses were performed with a maintained masking.

### Interventions

Yijinjing Qigong Exercise (YJJQE) and STE ran concurrently to minimize seasonal influences on disease activity. We encouraged participants to maintain their regular daily activities and to not start any new exercise programs. Both groups received educational information about the daily precautions, the knowledge of the physiology and pathology of KOA, and the importance of physical activity and home practice. A 12-week intervention program, with a frequency of twice a week, was provided to patients with KOA. Each exercise session included a 5 min warm-up, a 30 min practice, and a 5 min cool-down period. The intervention protocol included three distinctive phases, with the first phase (Weeks 1–2) focusing on fundamental principles, movement techniques, and safety precautions. The second phase (Weeks 3–4) focused on learning and practicing forms with their associated movements. The third phase (Weeks 5–12) emphasized completion of the family exercise plan independently by instructors to review video and homework materials. The instructors were required to have more than 5 years of professional experience. Training intensity was assessed using the modified Borg Fatigue Rating Scale, with a score of ≥5 as qualified (15).

#### YJJQE Group

**Figure 1:**
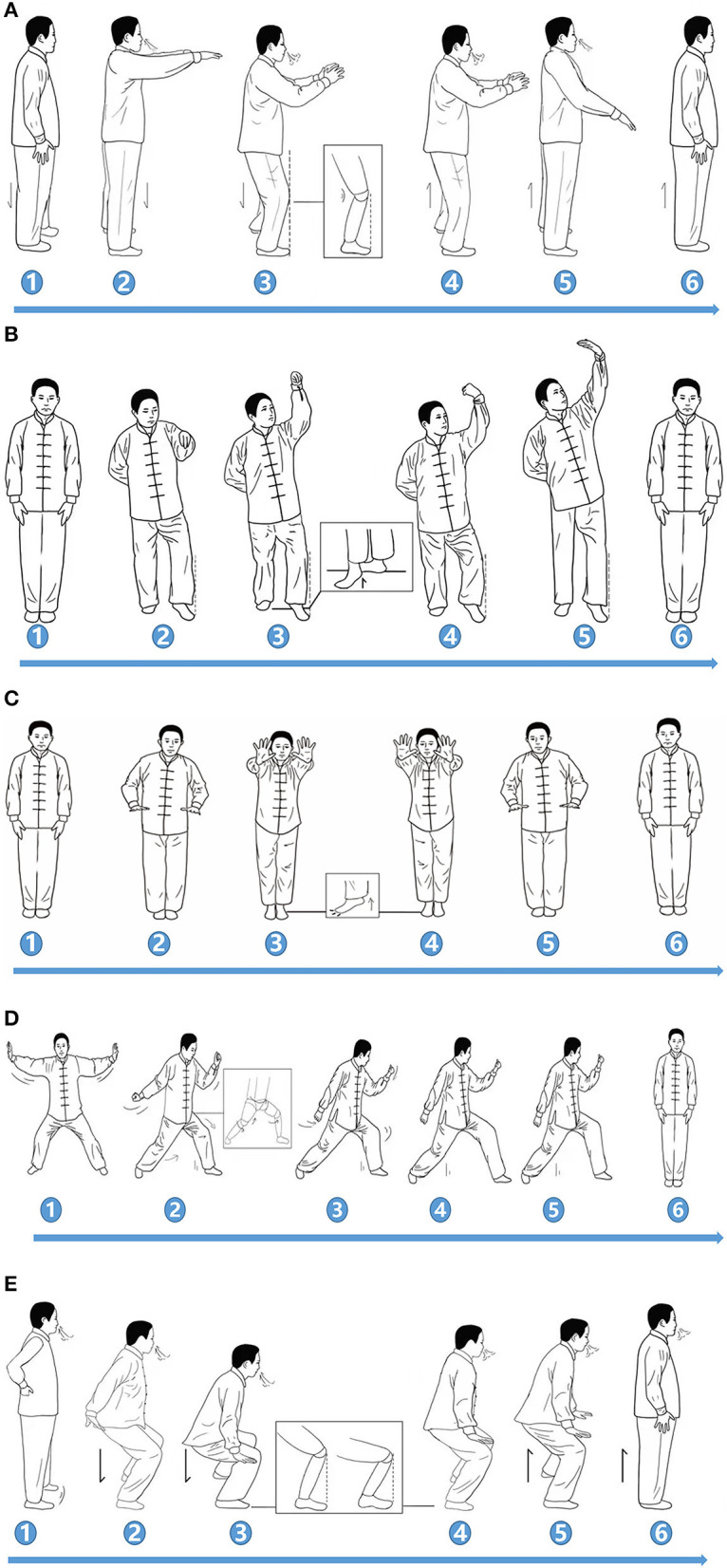
Demonstration of YJJQE. The figure was a schematic diagram of five actions, mainly including knee flexion, timing of exhalation and inhalation, and the direction of eye observation. **(A)** Movement: Place both elbow joints flexed in front of the chest, Bilateral flexion (2,3), and extension (4,5) of the knee joint. Breathing rhythm: Inhalation (2,3), Exhalation (4,5). Meditation: Staring at the floor 3 m ahead (2,3), focus on the position of the knee joint (4,5). **(B)** Movement: Extend one side of the upper limb to 45° outside of the head, Standing on one leg with knee flexed (2,3), with extended (4,5). Breathing rhythm: Inhalation (2,3), Exhalation (4,5). Meditation: Staring at the raised palm (2,3), focus on the position of the knee joint (4,5). **(C)** Movement: Both heels off the ground, toes grasping, both arms extended forward (2,3). Both heels fall to the ground with both upper limbs flexed backwards (4,5). Breathing rhythm: Inhalation (2,3), Exhalation (4,5). Meditation: Staring at the floor 3 m ahead (2,3), focus on the position of the knee joint (4,5). **(D)** Movement: Lateral lunge, center of gravity forward foot (2,3), Lateral lunge, the center of gravity moves backward and the hip joint rotates outward (4,5). Breathing rhythm: Inhalation (2,3), Exhalation (4,5). Meditation: Staring at the floor 3 m ahead (2,3), focus on the position of the knee joint (4,5). **(E)** Movement: Press both hands down on both sides of the knee joint with knee flexion (maximum Angle) (2,3), with knee extension flexion (maximum Angle) (4,5). Breathing rhythm: Inhalation (2,3), Exhalation (4,5). Meditation: Staring at the floor 3 m ahead (2,3), focus on the position of the knee joint (4,5).

The exercise protocol consists of five Qigong operation processes compiled by the State Sports General Administration of China (16); the specific action characteristics of which are shown in Table 1. The overall training protocol followed an easy-to-difficult progression that focused on the center of the body mass, shift of garment, moderate knee flexion, extension and rotation movement, and coupled with breathing exercises, generally at the movement of knee flexion with inhalation. The entire YJJQE movements were closely integrated with rhythmic breathing and mindfulness meditation of scanning awareness of the knee movement (Figure 1).

**Table 1:** Details of the operation of Yijinjing Qigong Exercise (YJJQE).

**Sequence of actions**	**Action details**
Action 1	The subject slowly squats and puts his palms together in front of his chest. While inhaling, he slightly flexes the knee joints, keeping the knees not exceeding the toes as a suitable squatting angle. Keep the eyes on the ground about 3 m in front of your body. Concentrate and maintain the above position for 10 s. Then exhale while slowly straightening the knee joint. During this process, the eyes are closed tightly, and the participant feels the position of the knee joint. The operations at this stage also lasts for 10 s.
Action 2	The subject adopts a posture of standing back and forth with both feet, with the toes of the front feet on the ground, and the center of gravity on the other foot that is back. Place one hand 30 cm above the side of the head, and place the other hand on the lumbosacral area, and then perform knee flexion and extension with inhalation and exhalation. The rhythm is the same as Action 1. Keep his eyes on the fingertips when flexing the knees, and close his eyes when extending the knees which can focus on feeling the position of the knee joint.
Action 3	The subject stand upright, raised his arms forward and inhaled, his heels slowly lifted off the ground, and the toes were held on the ground. While extending the knees, the subject looked at the ground about three meters in front and below, stabilized his center of gravity, maintained balance for 10 s; then, while flexing the upper arm, the heel slowly drops, and he also need to feel the position of the knee joint.
Action 4	Stand with the lower limbs separated by a distance of 1 m, and extend the upper limbs to the limit in the horizontal direction. Then make a lunge with both lower limbs in the left and right directions, with the center of gravity on the forefoot, while inhaling, staring at the floor 3 m ahead for 8–10 s. Then the hip joint of the front lower limb is externally rotated, the knee joint is extended, and the knee joint of the rear lower limb is flexed, the center of gravity is on the hind foot, and the exhale is also maintained for 10 s, and finally restored to the initial preparation stage posture.
Action 5	Put his hands in front of the chest, and then flex his knee joint with inhalation (the range of flexion angle is larger than any other movement), put hands on the outside of the knee joint, holding for 2 s, and keep the eyes on the ground 3 m ahead; Then exhale, straighten the knee joint, close the eyes, feeling the position of the knee joint.

#### STE Group

**Figure 2:**
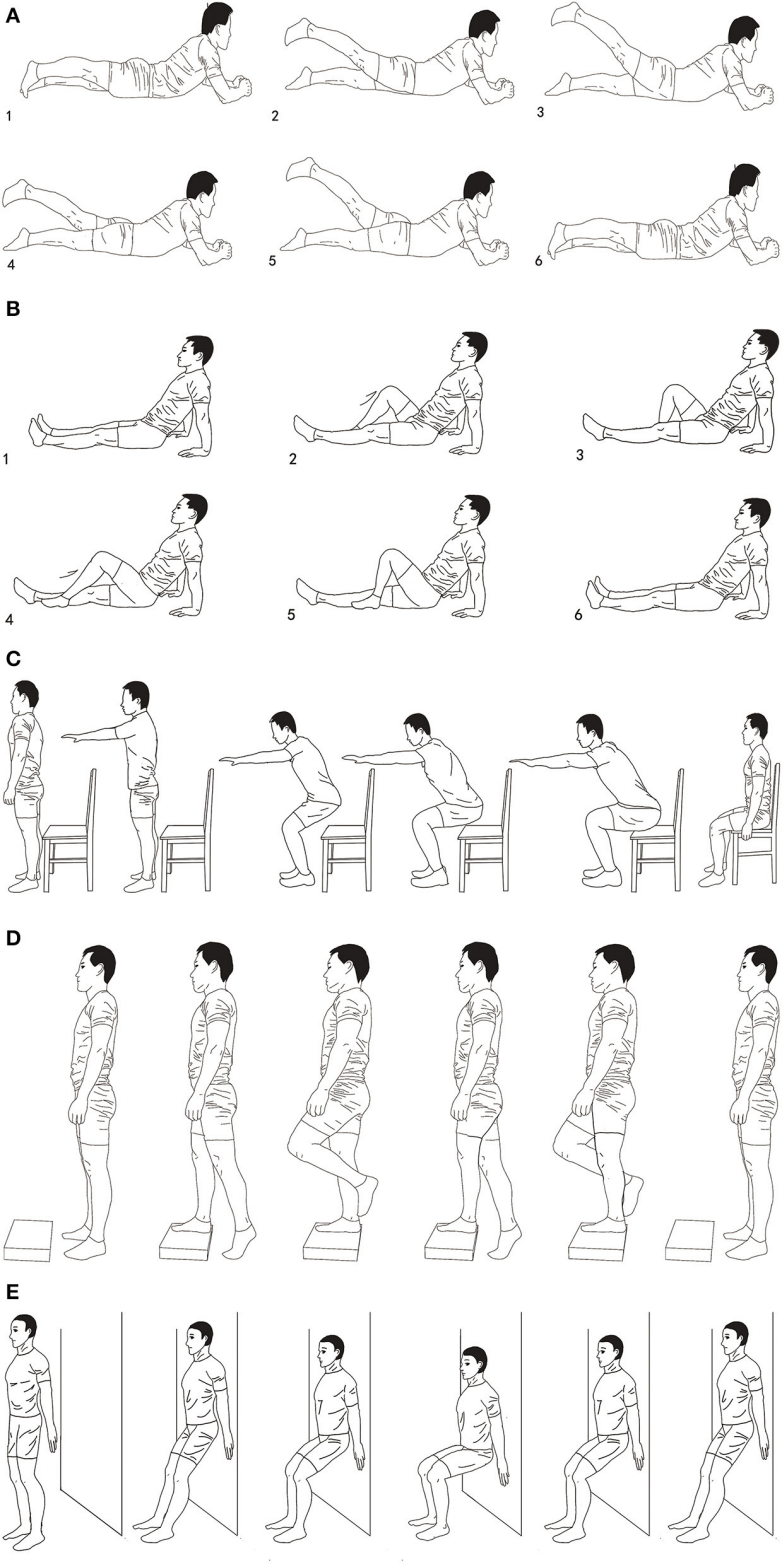
Demonstration of stretching training exercise (STE). The figure mainly described the stretching exercise flow of knee joint flexion and extension in different postures. **(A)** Lower limb flexion and extension in supine position, **(B)** lower limb flexion and extension in prone position, **(C)** sit and stand test training, **(D)** alternating one-legged stair support tasks, **(E)** static squatting training against the wall.

The prescription of STE consisting of quadriceps training and neuromuscular training is shown in Table 2. The five specific items of the program include lower limb flexion and extension while in supine position, lower limb flexion and extension while in prone position, sit and stand test training, alternating one-legged stair support tasks, and static squatting training against the wall, as recommended by the guideline (17) (Figure 2).

**Table 2:** Details of the operation of stretching training exercise (STE).

**Sequence of actions**	**Action details**
Action 1	The subjects are seated on the ground with both upper limbs supporting the ground for balance, and both lower limbs are placed horizontally in front of the ground. Then, the knee joint of one lower limb slowly centripetal flexed to 90°, held for 1–2 s, and then returned to the initial position. Contralateral leg stretching is the same.
Action 2	Subjects are in prone position with both elbows supporting their entire body weight. Both lower limbs are extended backward to the extreme position. Then one lower limb is supported and the opposite lower limb is extended backward and upward.
Action 3	Subjects performed the sit-stand alternating task with the help of a chair.
Action 4	Subjects performed alternating one-legged stair support tasks
Action 5	Subject's back is pressed against the wall to maintain static squat task, knee flexion is about 120°

### Outcome Measurements

All outcomes are collected at the baseline and at the end of the 12-week intervention period by the researchers.

#### Primary Outcome Measurement

##### Western Ontario and McMaster Universities Osteoarthritis Index (WOMAC)

It mainly includes three aspects, namely, pain, stiffness, and joint function to evaluate the structure and functional state of the knee joint. This approach is widely used in clinical evaluation of KOA symptoms with high reliability and validity (18).

#### Secondary Outcome Measurement

Secondary outcome measures include pain degree, quality of life, joint function, psychological evaluation, kinetic, and kinematic of human gait.

#### Visual Analog Scale

The visual analog scale (VAS) is a 10-point scale selected to quantitatively measure the level of osteoarthritis pain during the study, for which 0 indicates “no pain,” while 10 represents “unbearable pain” (19).

#### Short-Form 36 Item Health Survey Questionnaire (SF-36)

The SF-36 scale provides a comprehensive assessment of the overall quality of life of patients with KOA, which can be divided into two categories: physical component summary (PCS) and mental component summary (MCS), in accordance with the item attributes. The PCS score is the average of the scores of the first four dimensions (physiological function, physical function, physical pain, and general health) added together, while MCS is the average of the scores of the last four dimensions (emotional function, social function, mental health, and vitality) (20).

#### Beck Depression Inventory

Beck Depression Inventory (BDI) consists of a 21-question and validated self-evaluation scale tool used to measure the severity of depressive symptoms (21).

#### Perceived Stress Scale

Perceived Stress Scale (PSS) is mainly composed of 14 questions, a validated self-evaluation tool used to measure the severity of stress; the higher score reflects the increasing severity of symptoms (22).

#### Berg Balance Scale

The balance of the patients is mainly measured by evaluating the performance in 14 functional tasks including the following: from sitting to standing test, standing without support, circle test, step test, and standing on one foot (23).

#### Gait Analysis

Gait analysis could be used to realize the kinematics and dynamics of the lower limbs of patients with KOA; it can reflect the pathomechanical mechanism of patients (24). The following are the test procedures: (1) The participants were required to walk on a 10-m walkway in the laboratory, with three one-way walking tasks for each test. (2) The 3D marker trajectories were recorded at 100 Hz using a Vicon 16-camera MX13+ motion system (Vicon, Oxford Metrics Ltd., Oxford, UK). (3) A total of 28 marking points were pasted for body surface marking according to the anatomical position specified by the pyCGM2-LowerLimbCGM23 model. (4) Two force platforms (1200 Hz, Advanced 130Mechanical Technology, Inc., Watertown, MA, USA) were used to collect 3D kinematic data using Vicon Nexus software (Vicon Motion Analysis, Inc., Oxford, UK).

#### Safety Evaluation

Adverse events were recorded by participants through a case report form, defined as any problem they believed to be caused by an exercise program that required seeking treatment and/or that lasted more than 3 days, while drug (pain medications for KOA) combinations were recorded.

#### Sample Size Calculation

The sample size was calculated using the WOMAC pain scale as the primary outcome measurement. Previous research (25) showed that the mean value and standard deviation of the control group were 9.37 and 5.23, respectively, whereas the mean value and standard deviation of the intervention group were 5.15 and 3.24, respectively. Supposed that α = 0.05 and β = 0.10 and considering 20% rate of dropping out, the sample size was calculated according to the calculation formula. In the end, a total of 50 cases needed to be recruited, with 25 cases in each group.

#### Statistical Analyses

SPSS25.0 statistical software was used for statistical analysis by the independent party who was blinded to the group allocation. The *P*-values < 0.05 were considered significant. Shapiro–Wilk test was used to judge the normality of the data, and then the mean, standard deviation, maximum, minimum, median, credible interval, and frequency (composition ratio) were obtained. The primary result analysis would be an intention-to-treat analysis, where all participants were analyzed in the group and the missing data were imputed for three patients in the YJJQ group, and also for four patients in the STE group, using single mean or median interpolation. For continuous outcome measures, ANCOVA was used to adjust baseline outcome values to compare differences in mean change between groups (follow-up minus baseline). Results are presented as estimated differences with 95% confidence intervals (95% CIs). The likelihood of improvement in pain and function was compared between groups using log-binomial regression. Results are presented as relative risks with 95% CIs. Multivariate analysis was used to adjust for confounding factors.

## Results

**Figure 3:**
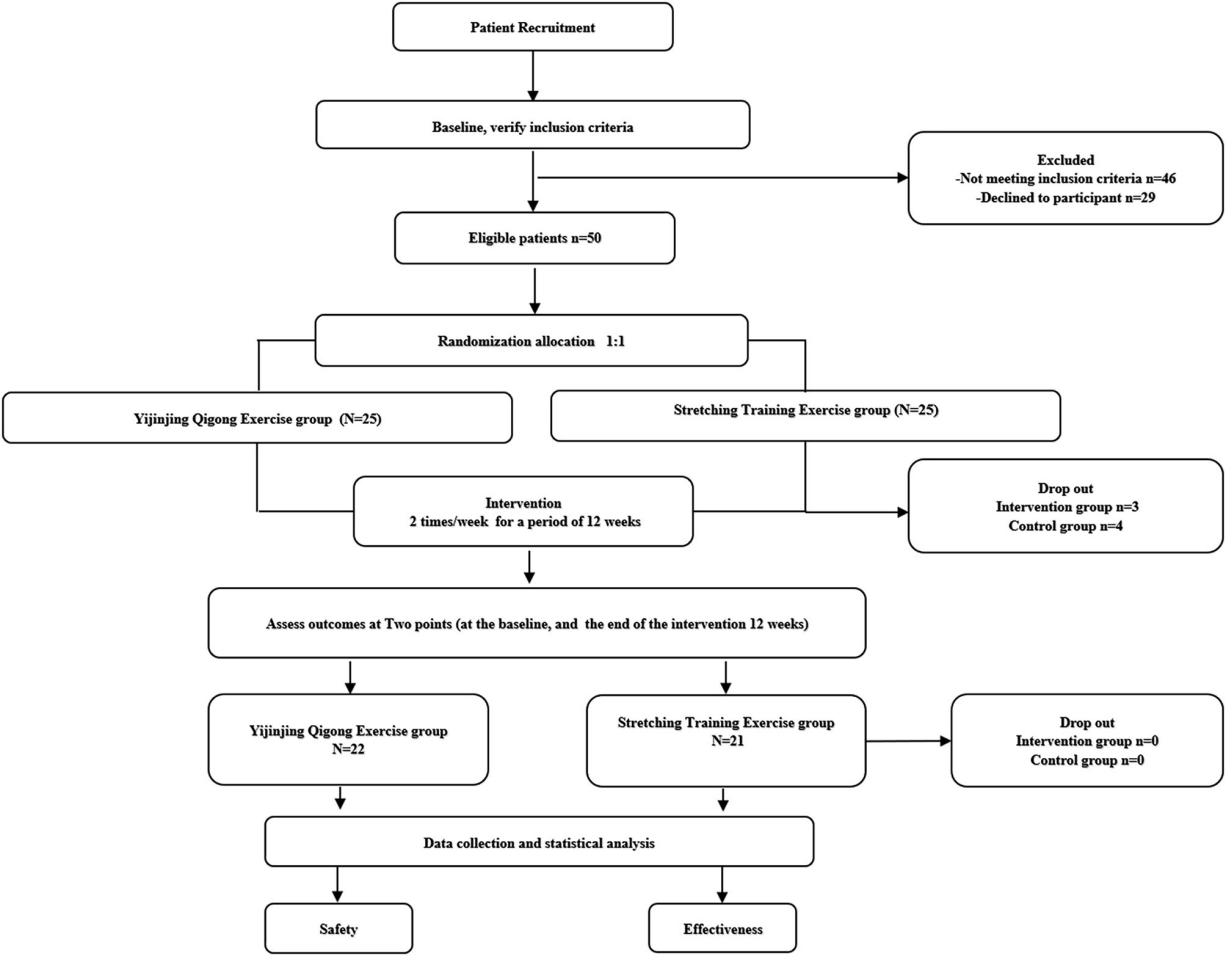
The flow of the trial.

Among the 125 participants, 75 (60%) did not satisfy the inclusion criteria or were unwilling to participate in the clinical trial. A total of 50 subjects with KOA were randomly assigned in a 1:1 ratio to the YJJQE group and to the STE group. The result showed no difference between the two groups in terms of demographic and characteristics of baseline data (*P* > 0.05, Table 3). The entire trail flow is shown in Figure 3, where 43 patients in the two groups completed the entire procedure while seven patients dropped out; three and four cases were disqualified in the YIIQE group and STE group, respectively, due to worsening symptoms. All participants attended at least 68 compliance diary sessions, and 88% of the participants attended all sessions; the data were comparable between groups, as shown in Table 3. Use of pain medications was similar across groups, without serious safety issues on either exercise regimen. However, generally, minor and transient problems were reported by some participants in the STE group [*n* = 12 (48%) participants] than in the YJJQE group [*n* = 6 (24%), *p* = 0.04] as increased muscle soreness [15 (60%) vs. 10 (40%)] in each group, respectively.

**Table 3:** Demographic and clinical characteristics of the YJJQE and STE groups^*^.

**Characteristic**	**YJJQE group** **(*n* = 25)**	**STE group** **(*n* = 25)**	***P***
Age, years	55.76 ± 8.37	53.40 ± 10.66	0.38
Height, cm	163 ± 6.84	166 ± 7.14	0.82
Body mass, kg	62.8 ± 10.49	64 ± 10.48	0.69
Body mass index, kg/m^2^	23.5 ± 3.23	22.9 ± 2.98	0.51
Males/Females (n)	4/21	9/16	0.107
Exercise compliance (number of completions, n)	76.56 ± 4.79	75.28 ± 5.43	0.382
Manual labor/non-manual labor (n)			
Current pain medication use, n (%)^&^	17 (68%)	18 (72%)	0.556
Non-steroidal anti-inflammatory drugs	14 (56%)	17 (72%)	0.239
Topical anti-inflammatory drugs	14 (56%)	15 (64%)	0.733
Adverse events	6 (24%)	12 (48%)	0.04

* *Indicated values are the mean ± SD and P < 0.05 was considered statistically significant difference. &: at any time; Adverse events were defined as any treatment-related problem that lasted for 3 days. YJJQE, Yijinjing Qigong Exercise; ST, Stretching Training Exercise*.

Between groups, albeit there were no differences between the YJJQE and STE groups for changes in the WOMAC pain score [mean difference 1.16 units (95% CI −0.46, 2.78)], WOMAC stiffness score [mean difference 0.2 units (95% CI −0.72, 1.12)], or WOMAC function score (0.32 units; −3.2, 3.84), no evidence of between-group differences for changes in any of the secondary outcomes was found, except the MCS score, BDI score, or PSS score, where the YJJQE group improved the unit values more significantly (*P* = 0.002, *P* = 0.001, and *P* = 0.026, Table 4). The application of the linear regression to adjust gender factors has shown consistent results (Additional file1). Also, no significant difference was found in the improvement of overall pain and dysfunction between the two groups. Improvement in pain was reported by 17 (68%) of 25 patients in the YJJQE group and 15 (60%) of 25 patients in the STE group (relative risk 1.41 [95% CI 0.44, 4.52], *p* = 0.56). Improvement in function was reported by 13 (52%) of 25 patients in the YJJQE group and 15 (60%) of 25 patients in the STE group (relative risk 0.72 [95% CI 0.23, 2.21], *P* = 0.57).

**Table 4:** Outcome measures in the YJJQE and STE groups^*^.

**Outcome**	**Week 0**		**Week 12**		**Within-group difference, week 12 minus week 0, mean (95% CI)#**		**Between-group difference, YJJQE minus STE**,
	**YJJQE** **(*N* = 25)**	**STE** **(*N* = 25)**	**YJJQE** **(*N* = 22)**	**STE** **(*N* = 21)**	**YJJQE** **(*N* = 22)**	**STE** **(*N* = 21)**	**mean (95% CI)#**
WOMAC pain score (range 0–50)	24.24 ± 3.07	23.68 ± 2.26	11.6 ± 1.44	12.2 ± 1.38	12.64 (11.32–13.96)	11.48 (10.38–12.57)	(1.16) (−0.46–2.78)
WOMAC stiffness score (range 0–20)	13.08 ± 1.22	13.72 ± 1.74	6.16 ± 1.49	7 ± 0.81	6.32 (5.66–6.98)	7.84 (6.95–8.73)	0.2 (−0.72–1.12)
WOMAC joint function score (range 0–170)	81.2 ± 6.27	79.84 ± 3.84	64.36 ± 5.13	63.32 ± 3.93	16.84 (14.53–19.16)	16.5 (14.81–18.22)	0.32 (−3.2–3.84)
VAS score for pain on walking	6.8 ± 0.71	7.08 ± 0.64	3.4 ± 0.91	2.96 ± 0.97	3.68 (3.25–4.10)	3.84 (3.34–4.34)	(−0.16) (−0.8–0.51)
MCS score	31.00 ± 6.38	32.75 ± 6.05	59.21 ± 9.04	47.08 ± 7.65	34.58 (30.22–38.95)	23.96 (20.15–27.78)	(−10.63) ^&^ [−17.07–(−4.19)]
PCS score	24.62 ± 6.40	23.12 ± 3.97	53.14 ± 6.64	54.61 ± 4.64	22.13 (18.78–25.49)	21.85 (19.13–24.57)	(−0.27) (−4.3–3.8)
BDI score	17.32 ± 2.15	18.48 ± 4.13	8.72 ± 1.42	13.4 ± 2.10	8.6 (7.6–9.5)	5.08 (3.4–6.8)	3.52^&^ (1.59–5.44)
PSS score	44.64 ± 1.57	45.36 ± 1.52	40.48 ± 1.61	42.92 ± 2.1	4.16 (3.07–5.24)	2.44 (1.36–3.51)	1.72^&^ (0.22–3.21)
BBS score	36.48 ± 2.04	35.84 ± 1.17	40.4 ± 3.20	39.2 ± 3.46	3.92 (2.52–5.31)	3.36 (2.1–4.59)	(−0.56) (−1.72–0.6)
Stride velocity (m/s)	0.71 ± 0.10	0.72 ± 0.11	1.21 ± 0.11	1.08 ± 0.09	0.49 (0.47–0.51)	0.5 (0.43–0.57)	0.003 (−0.06–0.07)
Stride length (m)	0.59 ± 0.04	0.57 ± 0.04	0.64 ± 0.03	0.64 ± 0.00	0.04 (0.02–0.07)	0.06 (0.04–0.08)	0.11 (0–0.02)
Stance phase time (s)	0.31 ± 0.05	0.31 ± 0.05	0.25 ± 0.09	0.26 ± 0.04	0.06 (0.02–0.1)	0.04 (0.01–0.08)	0.01 (−0.04–0.72)
Swing phase time (s)	0.36 ± 0.02	0.35 ± 0.06	0.38 ± 0.07	0.40 ± 0.06	0.01 (−0.05–0.01)	0.04 (−0.08–0)	0.02 (−0.02–0.06)

* *Indicated values are the mean ± SD. 95% CI, 95% confidence interval; &: P < 0.05; #: Adjusted for baseline values. VAS, visual analog scale; WOMAC, Western Ontario and McMaster Universities Osteoarthritis Index; MCS, Mental Component Summary; PCS, Physical Component Summary; BDI, Beck Depression Inventory; PSS, Perceived Stress Scale; BBS, Berg Balance Scale*.

## Discussion

Chronic pain of KOA often coexists with symptoms of anxiety and depression; it is a neuroscience problem that needs to be solved worldwide (26). The present study has focused on the effects of YJJQE on the pain, quality of life, and function for patients with KOA. This research showed the evidence that YJJQE, a mind-body exercise therapy, may potentially provide benefits over those of STE for the evaluation of mood disorders. Evidence of systematic review for Qigong has shown to improve depression (27, 28), and this study provided corresponding evidence for YJJQE. This finding might benefit from the fact that psychosomatic therapy has the dual mechanism of exercise and meditation that can relieve KOA pain and mood disorders (29, 30). The underlying mechanism that physical exercise, combined with continuous cognitive challenge training, can maintain a new neuron pool experiences, more complex cognitive tasks, and even recruits new neurons to improve brain function (31). A functional magnetic resonance study has shown that the functional connectivity of dorsolateral prefrontal cortex with left hippocampus and amygdala was enhanced in Qigong group. Therefore, the psychosomatic therapy can regulate the default network, amygdala, and also reward circuitry of emotion-related brain regions, while focusing on movement posture (32). Furthermore, the results showed that YJJQE and STE led to similar improvements in the WOMAC pain and function over 12 weeks. The five movements that adopted YJJQE can exercise the quadriceps muscle strength, improving the balance control ability and the proprioception, and is consistent with the previous results of traditional Qigong exercise (33). We have previously launched the clinical application of Taijiquan Qigong in elderly people with KOA, and the results showed that it can improve proprioception, knee motion, and pain symptom (25, 34, 35). The YJJQE movement operation is easier to learn and promote than the Taijiquan studies. In addition, a combination of quadriceps and neuromuscular stretching program was used as a control intervention, which was commonly prescribed for patients with KOA because it can reduce pain and improve function with a series of dynamic maneuvers of increased complexity (36, 37). Gait characteristics in KOA patients were pathologically abnormal in speed and in other spatiotemporal parameters (38). This study confirmed that YJJQE could improve the walking speed and restore the trend of normal gait, and also further kinematic and kinetic mechanisms continue to be published. Although no serious adverse reactions occurred, the STE group experienced more muscle soreness, where the dosage control in exercise prescription should be investigated in the follow-up study. Advantages of this study included a randomized controlled design and attention to key methodological features to compare the clinical efficacy of YJJQE and STE in patients with KOA. However, there are some limitations in this experiment. The physical therapists cannot be easily grouped blindly, but we asked the therapist to intervene with both groups, and the guidance was designed in advance to ensure the homogeneity of the therapist and intervention. We did not have a no-exercise control group and no significant difference in the primary outcome measurement, thus, between-group exercise effects should be interpreted with caution. The subjects were also followed up for 3 months, but the finding is not covered in this report. Although YJJQE can provide certain clinical evidence, it still cannot recommend specific exercise prescription dose.

## Conclusion

After 12 weeks of regular exercise therapy with YJJQE, there were no significant difference between the two groups of the primary outcome measurement (WOMAC) score, in other words, the effect of relieving pain and of improving dysfunction was the same as STE. However, the secondary outcomes showed an advantage over STE on the improvement of mood disorders, such as anxiety, depression, and mental health score.

## Data Availability Statement

The original contributions presented in the study are included in the article/Supplementary Material, further inquiries can be directed to the corresponding authors.

## Ethics Statement

The studies involving human participants were reviewed and approved by Ethics Committee of Yue Yang Hospital of Integrated Traditional Chinese and Western Medicine affiliated with Shanghai University of Traditional Chinese Medicine. The patients/participants provided their written informed consent to participate in this study.

## Author Contributions

SZ, GG, XL, FY, ZW, QZ, and MF drafted, reviewed, and revised the manuscript. QZ and MF managed the methodology of the study. All authors have read and approved the final manuscript.

## Funding

This study was financially supported by the Project of National Key Research and Development Program of Modernization Research of Traditional Chinese Medicine (2018YFC1707800), Qihuang Scholar (2018), National Natural Science Foundation of China (8030121, 81973973), Budget Project of Shanghai University of Traditional Chinese Medicine (2020LK057). The funders had no role in the design of the study, analysis, collection, and interpretation of the data, or the writing and decision for publication of the manuscript.

## Conflict of Interest

The authors declare that the research was conducted in the absence of any commercial or financial relationships that could be construed as a potential conflict of interest.

## Publisher's Note

All claims expressed in this article are solely those of the authors and do not necessarily represent those of their affiliated organizations, or those of the publisher, the editors and the reviewers. Any product that may be evaluated in this article, or claim that may be made by its manufacturer, is not guaranteed or endorsed by the publisher.
