# Supplementary material for: The Effectiveness of Traditional Chinese Yijinjing Qigong Exercise for the Patients With Knee Osteoarthritis on the Pain, Dysfunction, and Mood Disorder: A Pilot Randomized Controlled Trial
Source: Front Med (Lausanne). 2022 Jan 11;8:792436. doi: 10.3389/fmed.2021.792436 (PMC8787110; doi:10.3389/fmed.2021.792436)
Supplement: Supplementary file 1 [file Data_Sheet_1.pdf]

Table 1 Multivariate analysis (linear regression) of gender associated with outcomes

| Dependent variable | Independent variable | B | Standard error | Beta | P | R2 | Adjust-R2 | Durbin-Watson |
|---|---|---|---|---|---|---|---|---|
| WOMAC pain score (range 0–50) | Gender | -1.272 | 2.95 | 0.084 | 0.331 | 0.046 | 0.005 | 1.378 |
| WOMAC stiffness score (range0–20) | Gender | -0.018 | 1.85 | -0.004 | 0.929 | 0.003 | -0.039 | 1.601 |
| WOMAC joint function score (range0–170) | Gender | -1.32 | 4.93 | -0.12 | 0.704 | 0.015 | -0.027 | 1.181 |
| VAS score for pain on walking | Gender | -0.469 | 1.11 | -0.186 | 0.401 | 0.038 | -0.003 | 1.915 |
| MCS score | Gender | 5.636 | 9.71 | 0.223 | 0.001 | 0.277 | 0.247 | 1.436 |
| PCS score | Gender | -1.277 | 7.44 | -0.007 | 0.867 | 0.006 | -0.036 | 1.946 |
| BDI score | Gender | -1.219 | 3.39 | -0.142 | 0.002 | 0.237 | 0.205 | 1.286 |
| PSS score | Gender | 0.702 | 2.62 | 0.114 | 0.04 | 0.114 | 0.076 | 1.833 |
| BBS score | Gender | -1.325 | 3.17 | -0.185 | 0.38 | 0.04 | 0.001 | 0.813 |
| Stride velocity(m/s) | Gender | -0.086 | 0.11 | -0.319 | 0.09 | 0.096 | 0.058 | 1.47 |
| Stride length(m) | Gender | -0.023 | 0.052 | -0.194 | 0.315 | 0.048 | 0.007 | 1.82 |
| Stance phase time(s) | Gender | 0.027 | 0.09 | 0.131 | 0.593 | 0.022 | -0.02 | 2.087 |
| Swing phase time(s) | Gender | 0.009 | 0.83 | 0.048 | 0.668 | 0.017 | -0.025 | 2.159 |
